# The Pathophysiology of H_2_S in Renal Glomerular Diseases

**DOI:** 10.3390/biom12020207

**Published:** 2022-01-26

**Authors:** Karl-Friedrich Beck, Josef Pfeilschifter

**Affiliations:** Pharmazentrum Frankfurt/ZAFES, Institut für Allgemeine Pharmakologie und Toxikologie, Goethe-Universität, 60590 Frankfurt am Main, Germany; pfeilschifter@em.uni-frankfurt.de

**Keywords:** hydrogen sulfide (H_2_S), gasotransmitters, glomerulus, mesangial cells

## Abstract

Renal glomerular diseases such as glomerulosclerosis and diabetic nephropathy often result in the loss of glomerular function and consequently end-stage renal disease. The glomerulus consists of endothelial cells, mesangial cells and glomerular epithelial cells also referred to as podocytes. A fine-tuned crosstalk between glomerular cells warrants control of growth factor synthesis and of matrix production and degradation, preserving glomerular structure and function. Hydrogen sulfide (H_2_S) belongs together with nitric oxide (NO) and carbon monoxide (CO) to the group of gasotransmitters. During the last three decades, these higher concentration toxic gases have been found to be produced in mammalian cells in a well-coordinated manner. Recently, it became evident that H_2_S and the other gasotransmitters share common targets as signalling devices that trigger mainly protective pathways. In several animal models, H_2_S has been demonstrated as a protective factor in the context of kidney disorders, in particular of diabetic nephropathy. Here, we focus on the synthesis and action of H_2_S in glomerular cells, its beneficial effects in the glomerulus and its action in the context of the other gaseous signalling molecules NO and CO.

## 1. Introduction

For quite a long time, H_2_S was solely recognized as a smelly and toxic gas, causing fatal poisoning Fatal poisoning in the processing industry and in bathing accidents in lakes where the thermocline, which is consequently the H_2_S-containing layer, is near the surface. Physiologically, H_2_S affects the cytochrome c oxidase in the mitochondrial electron transport chain. Likewise, nitric oxide (NO) and carbon monoxide (CO), were originally also known as poisonous gases and were characterized as physiologic signalling molecules only in the 1980s. However, a physiological role for H_2_S was been demonstrated for the first time in 1996, when Hideo Kimura’s group showed that H_2_S possesses physiological signalling properties as a neuromodulator [1]. In this excellent research paper, Abe et al. were able to demonstrate that H_2_S acts via the NMDA receptor enhancing hippocampal long-term potentiation. Later, on the basis of their similar structure and their similar properties as signalling molecules, the inorganic gases NO, CO and H_2_S were denoted as gasotransmitters [2]. Meanwhile, great efforts in understanding the regulation of gasotransmitter synthesis as well as gasotransmitter-directed signalling were made, and the role of these signalling molecules in several diseases is currently in the focus of physiological and pharmacological research.

Kidney diseases are a severe global problem. Nutrition habits in the Western world and its changes also in developing countries shows a higher incidence of high blood pressure and type 2 diabetes, two major causes of kidney injury, resulting in many deaths and high expenses for the public health care systems. Besides diabetes, adverse drug effects, viral and bacterial infections or autoimmune disorders, such as systemic lupus erythematosus (SLE), often result in the development of glomerular kidney diseases. Frequent attendant symptoms of glomerulopathies are expansion of glomerular matrix, podocyte loss, followed by disability of the glomerular filtration barrier as well as proteinuria and glomerulosclerosis. As a consequence of diabetic glucose levels and other causes of glomerular injury, glomerular cells are affected by advanced glycation end products (AGEs), toxins, mechanical stress and reactive oxygen species (ROS). This stress situation interferes with the complex and fine-tuned intraglomerular crosstalk between mesangial cells, glomerular epithelial cells (podocytes) and glomerular endothelial cells resulting in a disturbance of glomerular integrity and function [3,4,5,6]. In the last decade, the research on H_2_S in animal models revealed promising results for the pharmacological use of H_2_S-releasing drugs for the treatment of glomerular kidney diseases [7]. Here, we briefly discuss the role of H_2_S in glomerulopathies with a focus on the molecular mechanisms of production and action of this signalling mediator.

## 2. Endogenous Synthesis of H_2_S

**Figure 1:**
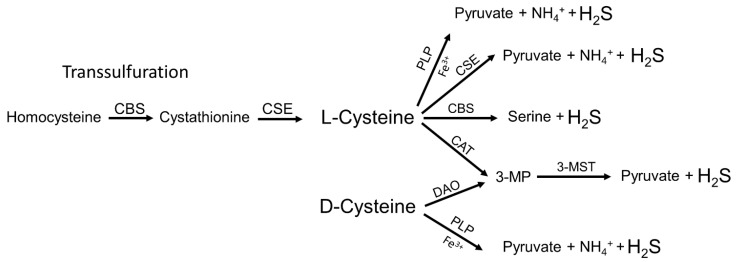
Enzymatic and non-enzymatic synthesis of H_2_S. The enzymatic synthesis of H_2_S by CSE, CBS, 3-MST and DAO as well as the non-enzymatic pathway catalysed by Fe^3+^ and PLP are presented. Please note that H_2_S is also formed by CBS and CSE through other steps of the transulfuration pathway [13]: This is a modification of Fig. 1C from Beck and Pfeilschifter [6]. CAT, cysteine aminotransferase; DAO, D-amino acid oxidase; 3-MP, 3-mercaptopyruvate; 3-MST, 3-mercaptopyruvate sulfurtransferase; CBS, cystathionine-β-synthase; CSE, cystathionine-γ-lyase; and PLP, pyridoxal phosphate.

H_2_S-synthesising enzymes such as cystathionine β-synthase (CBS), cystathionine γ-lyase (CSE) and 3-mercaptopyruvate sulfotransferase (3-MST) were characterized already in the 1940s and 1950s, but at that time the researchers were focussed on the characterization of the transulfuration pathway and they had not given much attention to its by-product, H_2_S [8]. To our knowledge, the detection of considerable amounts of H_2_S produced along the transulfuration pathway in liver and kidney tissues, that drove the hypothesis of a possible physiological role of endogenously produced H_2_S, was first described in 1982 in a highly cited paper by Stipanuk and Beck [9]. The amino acid L-cysteine is not only essential for protein synthesis, but it is also part of the tripeptide glutathione that is regarded as the most important physiological antioxidant formed in the body. Furthermore, thiols of cysteine residues in proteins serve as potent signalling devices by providing the target for thiol-based redox switches as induced by ROS, NO and H_2_S [10,11,12]. The only metabolic pathway serving for the endogenous synthesis of L-cysteine in mammalians is the transulfuration pathway [13]. Briefly, CBS forms cystathionine by the condensation of homocysteine with serine. Cystathionine is then further converted to L-cysteine by CSE. In turn, L-cysteine serves as a substrate for both CBS and CSE for the production of H_2_S. It is worth noting that H_2_S is not only formed from L-cysteine but also by the transulfuration pathway with L-homocysteine as a substrate for CSE. 3-MST is a further enzyme predominantly found in mitochondria that synthesizes H_2_S. It converts 3-mercaptopyruvate to pyruvate and H_2_S [14]. For this reaction, 3-mercaptopyruvate is provided by the enzymatic activity of cysteine aminotransferase (CAT) that converts L-cysteine and α-ketoglutarate to glutamate and 3-mercaptopyruvate. 3-mercaptopyruvate can also be formed by D-aminotransferase (DAO), which uses D-cysteine supplied with the daily diet [15]. Recently, also a non-enzymatic device for the generation of H_2_S has been described. Yang et al. demonstrate a direct reaction of L-cysteine with pyridoxal(phosphate) and iron resulting in the synthesis of pyruvate, ammonia (NH_3_) and H_2_S [16]. However, the physiological or pathophysiological relevance of this phenomenon has to be elucidated in further studies. Taken together, the synthesis of H_2_S occurs in a very complex manner by the action of different enzymatic and most probably also non-enzymatic mechanisms (Figure 1). In addition, the enzymes involved in H_2_S production are themselves controlled by transcriptional or translational processes. For example, CBS is regarded to be constitutively expressed at the transcriptional level, but its activity is strongly modulated by posttranscriptional mechanisms e.g., by NO and CO that affect the intrinsic haem group, resulting in decreased activity or by S-adenosylmethionine that acts as a positive regulator on CBS activity [17,18]. By contrast, CSE expression is induced at the transcriptional level by the transcription factor-specific protein 1 (Sp1), nuclear factor kappa B (NFκB) and nuclear factor erythroid 2–related factor 2 (Nrf2) [19,20,21].

## 3. H_2_S and Its Derivatives as Important Signalling Molecules

**Figure 2:**
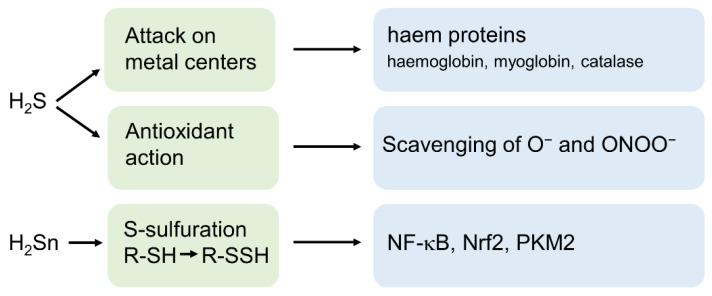
Molecular targets of H_2_S and polysulfides (H_2_Sn). H_2_S affects metalloproteins and acts as a scavenger of superoxide anion (O_2_^−^) and peroxynitrite (ONOO^−^). H_2_Sn is the most important mediator of S-sulfuration of cysteine thiols. NF-kB, Nuclear factor kappa-light-chain-enhancer of activated B-cells; Nrf2, Nuclear factor erythroid 2-related factor 2; PKM2, pyruvate kinase M2.

H_2_S exerts its toxic effects predominantly by attacking metal complexes such as the ferric iron of the mitochondrial cytochrome C oxidase and subsequent inhibition of mitochondrial synthesis of ATP [22]. Furthermore, it hampers oxygen transport in blood by affecting the ferrous iron of methaemoglobin, the reduced form of haemoglobin resulting in sulfhemoglobinemia [23]. Remarkably, besides its toxic effects, H_2_S-induced attacks on heme groups may also act as a regulatory signalling device [24]. Furthermore, H_2_S possesses important cytoprotective properties by its action as a potent antioxidant. Firstly, H_2_S is able to directly scavenge superoxide anions [25] and peroxynitrite [26] and acts consequently as a potent mediator against oxidative and nitrosative stress. Secondly, H_2_S triggers the expression of protective enzymes such as superoxide dismutases, catalase or glutathione peroxidase mainly by activating the redox-sensitive transcription factor Nrf2 [27] (Figure 2).

The third mechanism by which H_2_S directly modifies proteins is the formation of thiol-based redox switches on cysteine residues. Meanwhile, the conversion of cysteine thiols (-SH) to cysteine persulfides (-SSH), a process often referred to as S-sulfuration by H_2_S is regarded as the most relevant physiological mechanisms of H_2_S signalling [11,28]. The discovery that H_2_S (or rather its derivatives) directly affect the activity of a targeted protein established H_2_S besides ROS and NO, as a further important player in the orchestra of thiol-based redox switch-dependent cellular signalling devices [6,12]. At this point, it is important to note that due to the oxidation level of its sulfur atom (−2), H_2_S is not able to react directly with the sulfur of a cysteine thiol (with the same oxidation level) to produce persulfides. Meanwhile it is evident that polysulfides (H_2_Sn) also referred to as sulfane sulfur with oxidation levels of −1 or 0 are the crucial compounds that are responsible for S-sulfuration of thiols [29] (Figure 2). H_2_Sn is synthesized in the body by the activity of 3-MST or by partial oxidation of H_2_S by reactive oxygen or nitrogen species [29,30] and NO is considered as the most important mediator that reacts with H_2_S to produce H_2_Sn [31,32].

## 4. Physiology of Synthesis and Action of H_2_S in the Glomerulus

**Figure 3:**
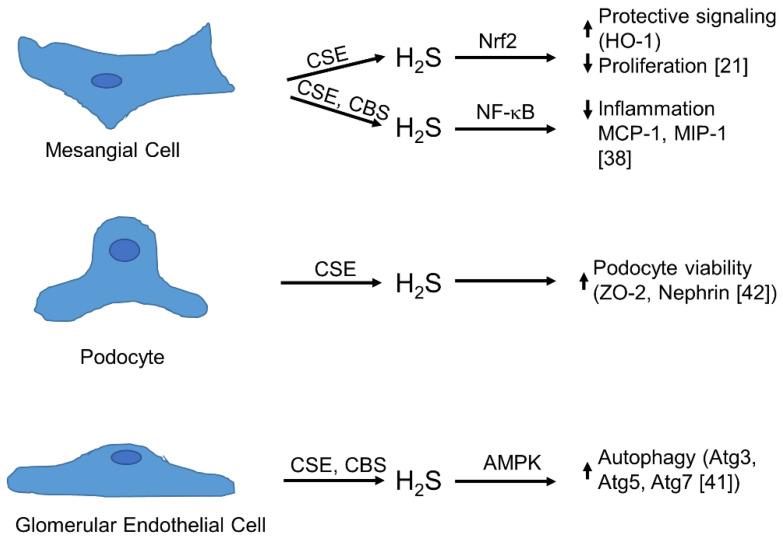
Synthesis and action of H_2_S in glomerular cells. Glomerular mesangial cells, podocytes and endothelial cells express CSE and CBS to produce H_2_S, which affects different cellular targets. Note that the H_2_S-producing enzyme 3-MST was characterized in whole glomeruli [37], but the expression of 3-MST cannot, so far, be assigned to a special cell type. Atg, autophagy related; CBS, cystathionine-b-synthase; CSE, cystathionine-g-lyase; HO-1, haem oxygenase 2; 3-MST, 3-mercaptopyruvate sulfurtransferase; MCP-1, monocyte chemoattractant protein-1; MIP-1, macrophage inflammatory protein 1; ZO-2, Zonula occludens protein 2.

Several disorders, such as diabetic nephropathy (often referred to as diabetic kidney disease, and IgA nephropathy, or autoimmune diseases, such as lupus erythematosus, may cause symptoms of glomerular disease, such as haematuria, proteinuria or edema, that are classical clinical consequences of a disturbed glomerular filtration barrier. Mesangial cells, glomerular epithelial cells (podocytes) and glomerular endothelial cells communicate in coordinated crosstalk in the glomerulus. Meanwhile, besides the role of growth factors, chemokines and inflammatory cytokines, the impact of gasotransmitters such as NO, CO and H_2_S has been more and more recognized as an important facet in this complex signalling network. Therefore, it is evident that gasotransmitters and the well-coordinated control of their production plays a crucial role in the interaction of glomerular cells [3,6]. Glomerular mesangial cells are located in the glomerular extracellular matrix and are important for the structure of the glomerular capillary tuft and for the function of the glomerular ultrafiltration apparatus [3]. Various pathological conditions such as high blood pressure, hyperglycaemia or infections disturb this delicate balance and result in the activation of mesangial cells, followed by a massive production of cytokines and chemokines and subsequent infiltration of neutrophils and macrophages. In this inflammatory context, mesangial cells and immune cells produce excessive amounts of ROS and NO, which further affect the function and viability of all glomerular cells [5,33,34]. These inflammatory cascades may end in irreversible destruction of the glomerulus and subsequent renal failure. However, if this detrimental inflammatory process often along with uncontrolled proliferation of mesangial cells and massive formation of ECM is terminated spontaneously or by appropriate and duly pharmacologic intervention, glomerular function can be restored by removal of dispensable mesangial cells and degradation of excess ECM. Therefore, it is desirable to expand our knowledge regarding the detailed molecular signalling mechanisms that decide between healing and loss of function to develop tailor-made strategies for the treatment of glomerulopathies. The use of gasotransmitter releasing compounds, in particular H_2_S—due to its properties as an anti-oxidant—reveals promising aspects for the treatment of glomerular diseases [35,36]. The expression of CSE and CBS has been demonstrated in glomerular endothelial cells, podocytes and mesangial cells derived from different species [6]. Less is known about the occurrence of 3-MST in glomerular cells. However, using a non-hypothesis-driven approach by 2D protein electrophoresis, the existence of 3-MST has been shown in whole glomeruli extracts of diabetic KKY mice [37]. All these findings suggest that the three main H_2_S-producing devices are active within the glomerulus and it is obvious that the different spatial and temporal expression and activity of three different enzymes in three different neighboured cells of different origins may serve as a delicate regulatory mechanism for the synthesis of H_2_S under physiologic and pathologic conditions (Figure 3). The expression of CSE and CBS in mouse mesangial cells was first reported in 2011 [38]. Sen and colleagues observed a marked increase in H_2_S formation in mesangial cells that were transfected with expression vectors containing CBS or CSE that resulted in a reduced inflammatory situation as observed by lower expression levels of chemokines as exemplified for macrophage inhibitory protein 1 (MIP-1) and monocyte chemoattractant protein-1 (MCP-1). Endogenous expression of CSE as well as CBS protein was demonstrated by immunoblotting. CSE protein expression was also found in the rat mesangial cell line HBZY-1 [39]. Remarkably, CSE expression was downregulated if the cells were kept in high glucose-containing medium and reduced H_2_S production under high glucose conditions may be one explanation for the important role of H_2_S observed in diabetic nephropathy. In another study, the same group demonstrated the expression of both, CSE and CBS mRNA, in HBZY-1 cells [40]. In contrast to the reports mentioned above, we could demonstrate CSE mRNA and protein expression as well as CSE activity but not CBS expression in primary rat mesangial cells [21]. In this study, we showed that treatment of mesangial cells with platelet-derived growth factor BB (PDGF-BB) induced CSE expression and activity. In addition, we found that PDFG-BB-induced formation of ROS and subsequent activation of the redox-dependent transcription factor Nrf2 triggered the induction of CSE expression. By immunohistochemistry, we observed also a marked upregulation of Nrf2 that was paralleled by enhanced CSE expression in the glomeruli from nephritic rats in a model of anti-Thy-1-induced glomerulonephritis. This indicated that enhanced CSE expression in an inflammatory environment may also occur in vivo. In mouse glomerular endothelial cells, high glucose conditions reduce CBS and CSE mRNA and protein expression that was paralleled by a reduced expression of the autophagy related genes Atg3, Atg5 and Atg7, accepted as markers for autophagy [41] (Figure 3). In turn, NaSH induced autophagy and this was dependent on adenosine monophosphate-activated protein kinase (AMPK) activity. CSE is also expressed in mouse podocytes as documented by two independent reports. Interestingly, as with mesangial cells and glomerular endothelial cells, a downregulation of CSE under high glucose conditions can also be observed in mouse podocytes. Interestingly, the high glucose-dependent downregulation of nephrin and zona occludens protein 2 (ZO-2) that goes along with the downregulation of CSE could be rescued by NaSH supplementation [42]. Since nephrin and ZO-1 are commonly used as markers for podocyte viability, one can speculate that H_2_S serves as a protective device in glomerular epithelial cells. In cultured mouse podocytes, Lee et al. observed that inhibition of phosphodiesterase 5 by tadalafil reduced high glucose-stimulated matrix production [43]. Tadalafil also stimulated CSE expression and this was paralleled by the phosphorylation of AMPK. Inhibition of CSE activity also diminished AMPK phosphorylation, indicating that H_2_S is involved in this signalling mechanism. Remarkably, inhibition of NO synthesis also inhibited H_2_S production and AMPK phosphorylation, demonstrating a delicate crosstalk between NO and H_2_S as well as their generating enzymes. Taken together, all glomerular cells possess one or more enzymes that are able to produce H_2_S (Figure 3) and it is tempting to speculate that a balanced H_2_S generation in different glomerular cell types contributes to the intraglomerular cross-talk under physiological and disease conditions.

## 5. The Role of H_2_S in Glomerular Pathophysiology and Disease

### 5.1. Diabetic Nephropathy

Various kidney diseases of different origin are accompanied with glomerular injury which often results in the complete loss of kidney function. To our knowledge, the role of H_2_S is best characterized in diabetic nephropathy. Already in 2012, Csaba Szabo summarized the literature on H_2_S and diabetic nephropathy and came to the conclusion that H_2_S exerts detrimental effects in the onset of type 1 diabetes by augmenting β-cell death in pancreatic islets [44]. However, in the course of type 1 and type 2 diabetes, H_2_S exerts protective effects on endothelial dysfunction accompanied with high glucose levels [44]. In this context, it is important to note that H_2_S plasma levels are often reduced in diabetic kidney disease and other chronic kidney disorders; therefore, from a pharmacological view, the treatment of kidney diseases with H_2_S-releasing molecules could also be denoted as a substitution therapy [45]. Recently, it has been demonstrated that treatment of streptozotocin diabetic rats with the H_2_S donor NaSH ameliorated classical symptoms of diabetic nephropathy such as glomerular basement membrane thickening and mesangial matrix deposition. In addition, the authors observed diminished NFκB signalling and enhanced expression of protective genes after activation of the redox-sensitive transcription factor Nrf2 [46]. Diabetic nephropathy displays inflammatory and profibrotic symptoms and results in redox stress by enhanced ROS production and this goes along with the activation the renin-angiotensin system (RAS). Remarkably, administration of H_2_S-releasing agents affects all these processes, further indicating H_2_S as a protective signalling molecule [45]. Meanwhile, a protective role of the more novel H_2_S donors GYY 4137 and S-propargyl-cysteine, also referred to as ZYZ-802, has been demonstrated in diabetic nephropathy using diabetic Akita mice and a model of streptozotocin-induced nephropathy, respectively [47,48]. Treatment of Akita mice with GYY 4137 restored reduced renal miR-194 expression and subsequently attenuated fibrosis. The anti-fibrotic effects of miR-194 were also confirmed in cultured glomerular endothelial cells using an miR-194 mimic [47]. Also the novel H_2_S donor S-propargyl-cysteine potently reduced fibrosis as observed by reduced mRNA expression of fibronectin and type IV collagen in streptozotocin-induced nephropathy. Moreover, in this animal model, S-propargyl-cysteine potently reduced inflammatory signalling processes [48].

### 5.2. Hyperhomocysteinemia-Induced Glomerular Sclerosis

**Figure 4:**
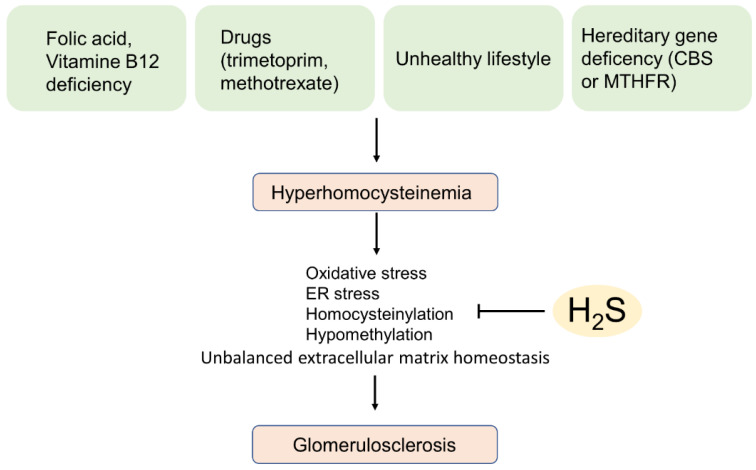
H_2_S attenuates hyperhomocysteinemia-induced glomerulosclerosis. H_2_S reduces symptoms hyperhomocysteinemia such as oxidative stress and ER-stress. CBS, cystathionine-β-synthase; ER stress, endoplasmic reticulum stress; MTHFR, methylenetetrahydrofolate reductase.

Since hyperhomocysteinemia occurs often in the context of lowered activity of the H_2_S generating enzyme CBS, it is worth dedicating a whole section on this disorder. Homocysteine is a non-proteinogenic amino acid that is a natural by-product of amino-acid metabolism. High plasma levels (above 15 µM) of homocysteine were recognized as important risk factors for the development of several disorders such as cardiovascular and kidney diseases as well as neurodegeneration [49,50,51]. Hyperhomocysteinemia arises from folic acid and vitamin B12 deficiency, adverse effects induced by drugs affecting the folic acid metabolism such as methotrexate or trimethoprim and by an unhealthy life-style with smoking, alcohol, overweight and physical inactivity. In addition, hyperhomocysteinemia is also a hereditary disorder that results from genetic defects of the genes for CBS or methylenetetrahydrofolate reductase (MTHFR), genes that metabolize homocysteine by transulfuration or remethylation, respectively [52,53] (Figure 4). High homocysteine levels decrease glomerular function by directly affecting glomerular cells by oxidative stress, endoplasmic reticulum stress, homocysteinylation and hypomethylation and consequently to a dysregulated extracellular matrix homeostasis, which finally may end-up in the development of severe glomerulosclerosis (reviewed in [54]). Remarkably, in a mouse model of hyperhomocysteinemia with a heterozygous depletion of the CBS gene (CBS ^(+/−)^ mice) it has been demonstrated that CBS ^(+/−)^ mice as expected show a lower expression of CBS but also of CSE and this was accompanied by reduced levels of H_2_S. Importantly, administration of the H_2_S donor NaSH with the drinking water in CBS ^(+/−)^ mice reduced hyperhomocysteinemia and glomerulosclerosis and normalized collagen deposition in renal cortical tissue [55,56]. In cultured mouse mesangial cells that were double transfected with expression vectors bearing the genes for CSE and CBS, the same group demonstrated a protective effect of H_2_S on homocysteine-induced inflammation [38]. Furthermore, H_2_S mitigates homocysteine-mediated apoptosis and matrix remodelling by Akt/FOXO1 signalling in mesangial cells [57]. In the mouse model of CBS ^(+/−)^ hyperhomocysteinemia, homocysteine thiolactone, a highly reactive homocysteine metabolite has been demonstrated to directly affect eNOS by N- homocysteinylation and consequently reducing the bioavailability of NO. Administration of the H_2_S donor NaSH reduced the symptoms of hyperhomocysteinemia and restored bioavailability of NO [58]. In summary, the therapeutic use of H_2_S-generating drugs may have a protective effect in hyperhomocysteinemia-related glomerular diseases.

### 5.3. Acute Kidney Injury

Patients in intensive care units that were hospitalized due to heart, liver or kidney complications are often affected by acute kidney injury (AKI). AKI is characterized by a rapid loss of renal function, that develops within hours or a few days. Besides initial conditions of illness as mentioned above, AKI may evolve from sepsis, different forms of glomerulonephritis, ischemia/reperfusion injury and adverse effects of drugs such as NSAIDs or cytostatic agents used for the treatment of cancer, in particular cisplatin [59]. Depending on its origin, AKI affects preferentially either glomerular or tubular structures of the kidney. A protective role of hydrogen sulfide in AKI on all kidney segments, including the tubules, is reviewed elsewhere [7,60,61]. The effects of AKI in the context of different forms of glomerulonephritis is, so far, poorly understood. However, the different observations in cultured glomerular cells and in a rat model of anti-Thy-1-induced glomerulonephritis mentioned before showing a tight regulation of the synthesis and action of H_2_S, strongly suggest a protective role, as well, for H_2_S in the course of different forms of glomerulonephritis. However, this aspect of H_2_S action has to be demonstrated using suitable animal models in the future.

## 6. The Role of H_2_S in the Context of Gasotransmitter Signalling by NO and CO

**Figure 5:**
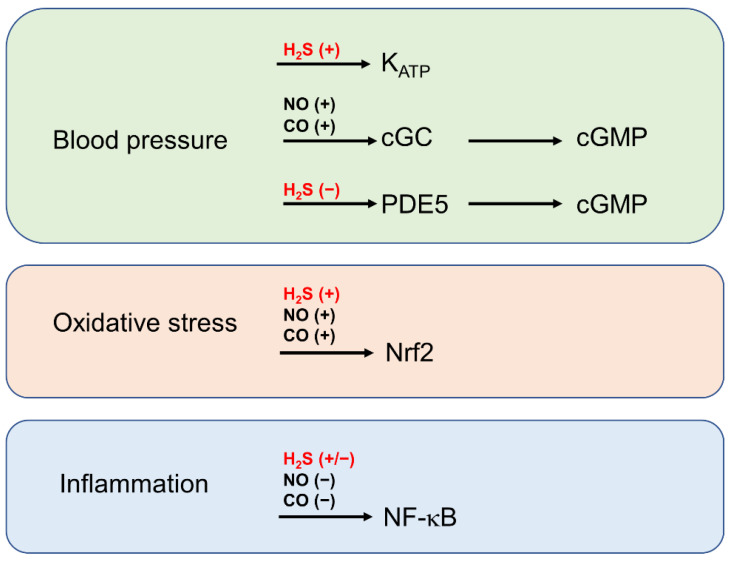
Possible effects of H_2_S on the classical symptoms of glomerular disease in comparison to other gasotransmitters. Gasotransmitters exert similar effects on blood pressure, oxidative stress and inflammation. Note that different effects of H_2_S-mediated S-transulfuration on NF-κB have been reported [64,65]. In contrast to NO and CO, H_2_S elevates cGMP levels not by activation of sGC, but by inhibition of PDE5. PDE5, phosphodiesterase 5; sGC, soluble guanylyl cyclase; cGMP, Cyclic guanosine monophosphate.

In recent decades, it became more and more evident that the three gasotransmitters NO, CO and H_2_S are important members of a complex redox signalling network. All these small molecules act on similar targets; therefore, it is not surprising that they are potentially able to replace each other under certain circumstances. It is particularly noticeable that NO, CO and H_2_S share not only common properties as anti-oxidants and vasodilators under physiological but also pathophysiological conditions. More recent research demonstrated that gasotransmitters also potently affect the gene expression pattern in a cell. In particular, the transcription factors NF-κB and Nrf-2 are meanwhile established molecular targets of gasotransmitters. Matthews and colleagues were the first that demonstrated the effect of a gasotransmitter on NF-κB activity. In an in vitro approach using recombinant NF-κB subunits p50 and p65, they found that the NO donors sodium nitroprusside (SNP) and S-nitroso-N-acetylpenicillamine (SNAP) potently S-nitrosated the p50 subunit at cysteine 62. They then found that this thiol-based redox switch resulted in a marked inhibition of NF-κB-binding activity as assessed by gel electrophoretic mobility shift assay (EMSA) [62]. Remarkably, NF-κB is also sensitive to S-nitrosation on the p65 subunit (cysteine 38) [63]. For their studies, they used respiratory epithelial cells and peritoneal macrophages as well as RAW 264.7 macrophages. Remarkably, S-nitrosation of p65 and subsequent inhibition of NF-κB-binding activity and NF-κB -mediated gene expression was achieved in cells forced to endogenously produce NO, demonstrating that—in comparison to the NO-donors used by Matthew et al.—physiological NO levels are sufficient to inhibit inflammatory signalling by the transcription factor NF-κB. The research on the role of H_2_S-induced thiol-based redox switches on NF-κB subunits and NF-κB activity appears more complex, leading to, at first glance, contradictory results. Interestingly, S-sulfuration at cysteine 38 of the p65 subunit of NF-κB was been shown to support NF-kB activity in murine macrophages by Sen and colleagues already in 2012 [64]. This finding supported the prevailing hypothesis at that time that S-nitrosation has a more inhibitory effect and S-sulfuration has a more activating effect on enzyme activity. However, the contrary effect has been demonstrated by Du et al. [65]. The authors reported that—also in murine macrophages—S-sulfuration of p65 at cysteine 38 attenuated translocation of NF-κB into the nucleus und subsequent reduction of NF-κB -dependent gene expression as exemplified for monocyte chemoattractant protein (MCP-1). These contradictory results reported in two independent publications suggest that the effects of thiol-based redox switches is dependent on a complex composition of different redox devices in a cellular environment. The third gasotransmitter, namely CO, is not able to directly affect proteins by the production of thiol-based redox switches, but it inhibits NF-κB signalling most probably by the synthesis of HO-1 via the Nrf2 pathway and subsequent S-glutathionylation of the p65 subunit [66]. Taken together, all gasotransmitters are able to exert anti-inflammatory effects by affecting NF-κB signalling, but this strongly depends on the specific redox environment (Figure 5). A second important target for gasotransmitter action is Nrf2. Nrf2 was originally discovered as a redox-dependent transcription factor in 1995 [67,68] and has been meanwhile recognized as a target of the gasotransmitters NO, CO and H_2_S. Thiol-based redox switches on Keap1, the natural inhibitor of Nrf2, have been first identified in pheochromocytoma (PC12) cells incubated with the NO donor SNAP [69] as well as in mouse embryonic fibroblasts in the presence of the H_2_S donor NaSH [70]. These modifications on Keap1 forced translocation of Nrf2 into the nucleus and both gasotransmitters induced classical target genes of Nrf2 such as heme oxygenase 1 (HO-1) or glutathione reductase, respectively. Remarkably, in a comparable manner with the NF-κB system mentioned above, NO and H_2_S share a common cysteine residue in Keap1 (Cys151) to produce redox-based thiol switches, indicating a common route for these gasotransmitters in Nrf2-mediated protective signalling processes. Also glomerular cells, namely human podocytes and human mesangial cells react with the H_2_S donors AP39, AP106, AP72, AP67 and GYY4134 with an enhanced expression of HO-1 that serves in many studies as a marker for Nrf2 activation. However, a direct interaction of H_2_S with the Keap1/Nrf2 system was not demonstrated in this report [71]. As already mentioned, CO is not able to form thiol-based redox switches. However, an activation of Nrf2-mediated gene expression has been demonstrated in human hepatocytes and in a mouse model of focal cerebral ischemia and this allows the conclusion that all gasotransmitters modify the gene expression pattern via the Keap1/Nrf2 axis into a protective direction [70,72] (Figure 5).

A third important signalling device, which is strongly affected by gasotransmitters and plays also a fundamental role in the regulation of gene expression is the soluble guanylate cyclase (sGC)/cGMP axis [73]. In glomerular cells, a protective role of NO in activating sGC and subsequent formation of cGMP has been recognized already 20 years ago [6,74,75]. To our knowledge, a role of another gasotransmitter, namely CO in the sGC/cGMP axis of the kidney, has only been demonstrated for the regulation of the tubulo-glomerular feedback [76].

Particularly in the vascular system, a protective role for CO and H_2_S by increasing cGMP levels is well understood. Morita and colleagues demonstrated, already in 1995, that CO endogenously generated along with enhanced HO-1 expression and activity elevates cGMP levels by a sGC-dependent mechanism in smooth muscle cells [77]. In contrast, H_2_S stabilizes cGMP by inhibition of cGMP-specific phosphodiesterases in aortic rings [78] and H_2_S supports NO-induced sGC activation as a reductant that forms bivalent (ferrous) iron in the haem moiety of sGC [79]. Generally, all gasotransmitters force the formation of cGMP, support protective anti-oxidant mechanisms by the activation of Nrf2-mediated gene transcription and—with some exceptions—inhibit inflammatory signalling processes by the inhibition of NF-κB (Figure 5). Not all of these issues mentioned in this chapter have so far been demonstrated in glomerular cells or in animal models of glomerular diseases. Nevertheless, cGMP, NF-κB and Nrf2 are important signalling molecules in glomerular cells and it is obvious that gasotransmitter-induced protective signalling is also evident in the renal glomerulus.

In addition to the concerted action of gasotransmitters on common targets, it is important to note that the synthesis of at least one enzyme for the synthesis of a certain gasotransmitter, namely the inducible NO synthase (iNOS), HO-1 and CSE are regulated by the redox-sensitive transcription factors NF-κB and Nrf2 and this may provide a direct control mechanism to maintain homeostasis of the gasotransmitter composition in a cell. The induction of iNOS by the transcription factor NF-κB has been demonstrated also in glomerular cells such as rat mesangial cells and mouse glomerular endothelial cells [80,81,82]. Remarkably, Nrf2 triggers the expression of both HO-1 and CSE in rat mesangial cells that were exposed to enhanced production of ROS after stimulation with PDGF-BB [21]. A more detailed description is given in [6] of NF-κB- and Nrf2-induced expression of gasotransmitter-producing enzymes in general and especially in glomerular cells.

## 7. Conclusions

Glomerular kidney diseases are usually treated with dietary reduction of salt intake, diuretics, vasodilating drugs such as AT-1 receptor antagonists or angiotensin-converting enzyme inhibitors. To address inflammatory symptoms, glucocorticoids, immunosuppressive drugs or anti-inflammatory biologics are widely used. Based on these well-established therapeutic strategies, the administration of H_2_S-releasing compounds or a regimen that forces the endogenous synthesis of H_2_S would represent an additional approach to treat a variety of glomerular kidney diseases. Due to its ability to directly activate K_ATP_ [83] channels or to elevate cGMP levels by the inhibition of PDEs [78], H_2_S acts as a powerful vasodilator (Figure 5). Furthermore, H_2_S is a potent anti-inflammatory agent. Therefore, it is obvious that pharmacological control of H_2_S-synthesis and action may support the treatment of glomerular diseases with other vasodilating or anti-inflammatory compounds. In addition, H_2_S acts as a direct anti-oxidant and targets Nrf2 to induce anti-oxidant/protective gene expression. Indeed, as shown in several animal models, Nrf2 inducing agents such as oltipraz or sulforaphane are able to alleviate symptoms of kidney diseases [84]. A promising activator of Nrf2, namely bardoxolone methyl, has been shown to improve the glomerular filtration rate in patients suffering from diabetic nephropathy. However, the possible appearance of cardiovascular problems in some patients should be taken seriously, before this drug can enter the market [85]. Taken together, H_2_S is a promising gaseous mediator, which possesses vasodilatory, anti-inflammatory and anti-oxidant properties. However, the effects of H_2_S are highly concentration-dependent and this has to be considered in the development of suitable H_2_S donors. Moreover, H_2_S affects a series of targets in the body that may result in severe adverse effects. For example, the effects of H_2_S on K_ATP_ channels in pancreatic b-cells may worsen the symptoms of diabetes [86]; therefore, more extensive research is needed to exclude such effects in the treatment of diabetic nephropathy with H_2_S donors. Several H_2_S-releasing molecules, many of them derivatives of nonsteroidal anti-inflammatory drugs such as ibuprofen or naproxen, are currently under investigation for the treatment of various diseases in animal models but also in clinical studies. Moreover, for the treatment of glomerular kidney diseases promising results from animal models do exist, but to our knowledge, so far, there are no results from clinical trials available [60]. Further studies are definitely needed to evaluate the role of H_2_S in glomerular kidney disease with a special focus on its interplay with other gasotransmitters and ROS.

## Data Availability

Not applicable.

## References

[1] Abe K., Kimura H. (1996). The Possible Role of Hydrogen Sulfide as an Endogenous Neuromodulator. J. Neurosci..

[2] Wang R. (2002). Two’s Company, Three’s a Crowd: Can H_2_S Be the Third Endogenous Gaseous Transmitter?. FASEB J..

[3] Schlöndorff D., Banas B. (2009). The Mesangial Cell Revisited: No Cell Is an Island. J. Am. Soc. Nephrol..

[4] Pfeilschifter J. (1989). Cross-Talk between Transmembrane Signalling Systems: A Prerequisite for the Delicate Regulation of Glomerular Haemodynamics by Mesangial Cells. Eur. J. Clin. Invest..

[5] Schlöndorff D. (2014). Putting the Glomerulus Back Together: Per Aspera Ad Astra (“a Rough Road Leads to the Stars”). Kidney Int..

[6] Beck K.-F., Pfeilschifter J. (2021). Gasotransmitter Synthesis and Signalling in the Renal Glomerulus. Implications for Glomerular Diseases. Cell. Signal..

[7] Ngowi E.E., Sarfraz M., Afzal A., Khan N.H., Khattak S., Zhang X., Li T., Duan S.-F., Ji X.-Y., Wu D.-D. (2020). Roles of Hydrogen Sulfide Donors in Common Kidney Diseases. Front. Pharmacol..

[8] Szabo C. (2018). A Timeline of Hydrogen Sulfide (H_2_S) Research: From Environmental Toxin to Biological Mediator. Biochem. Pharmacol..

[9] Stipanuk M.H., Beck P.W. (1982). Characterization of the Enzymic Capacity for Cysteine Desulphhydration in Liver and Kidney of the Rat. Biochem. J..

[10] Paget M.S.B., Buttner M.J. (2003). Thiol-Based Regulatory Switches. Annu. Rev. Genet..

[11] Longen S., Beck K.-F., Pfeilschifter J. (2016). H_2_S-Induced Thiol-Based Redox Switches: Biochemistry and Functional Relevance for Inflammatory Diseases. Pharmacol. Res..

[12] Paulsen C.E., Carroll K.S. (2013). Cysteine-Mediated Redox Signaling: Chemistry, Biology, and Tools for Discovery. Chem. Rev..

[13] Sbodio J.I., Snyder S.H., Paul B.D. (2019). Regulators of the Transsulfuration Pathway. Br. J. Pharmacol..

[14] Shibuya N., Tanaka M., Yoshida M., Ogasawara Y., Togawa T., Ishii K., Kimura H. (2009). 3-Mercaptopyruvate Sulfurtransferase Produces Hydrogen Sulfide and Bound Sulfane Sulfur in the Brain. Antioxid. Redox. Signal..

[15] Shibuya N., Koike S., Tanaka M., Ishigami-Yuasa M., Kimura Y., Ogasawara Y., Fukui K., Nagahara N., Kimura H. (2013). A Novel Pathway for the Production of Hydrogen Sulfide from D-Cysteine in Mammalian Cells. Nat. Commun..

[16] Yang J., Minkler P., Grove D., Wang R., Willard B., Dweik R., Hine C. (2019). Non-Enzymatic Hydrogen Sulfide Production from Cysteine in Blood Is Catalyzed by Iron and Vitamin B6. Commun. Biol..

[17] Taoka S., Banerjee R. (2001). Characterization of NO Binding to Human Cystathionine Beta-Synthase: Possible Implications of the Effects of CO and NO Binding to the Human Enzyme. J. Inorg. Biochem..

[18] Majtan T., Pey A.L., Kraus J.P. (2016). Kinetic Stability of Cystathionine Beta-Synthase Can Be Modulated by Structural Analogs of S-Adenosylmethionine: Potential Approach to Pharmacological Chaperone Therapy for Homocystinuria. Biochimie.

[19] Yang G., Pei Y., Teng H., Cao Q., Wang R. (2011). Specificity Protein-1 as a Critical Regulator of Human Cystathionine Gamma-Lyase in Smooth Muscle Cells. J. Biol. Chem..

[20] Wang M., Guo Z., Wang S. (2014). The Binding Site for the Transcription Factor, NF-ΚB, on the Cystathionine γ-Lyase Promoter Is Critical for LPS-Induced Cystathionine γ-Lyase Expression. Int. J. Mol. Med..

[21] Hassan M.I., Boosen M., Schaefer L., Kozlowska J., Eisel F., von Knethen A., Beck M., Hemeida R.A.M., El-Moselhy M.A.M., Hamada F.M.A. (2012). Platelet-Derived Growth Factor-BB Induces Cystathionine γ-Lyase Expression in Rat Mesangial Cells via a Redox-Dependent Mechanism. Br. J. Pharmacol..

[22] Nicholls P., Kim J.K. (1982). Sulphide as an Inhibitor and Electron Donor for the Cytochrome c Oxidase System. Can. J. Biochem..

[23] Ríos-González B.B., Román-Morales E.M., Pietri R., López-Garriga J. (2014). Hydrogen Sulfide Activation in Hemeproteins: The Sulfheme Scenario. J. Inorg. Biochem..

[24] Pietri R., Román-Morales E., López-Garriga J. (2011). Hydrogen Sulfide and Hemeproteins: Knowledge and Mysteries. Antioxid. Redox Signal..

[25] Al-Magableh M.R., Kemp-Harper B.K., Ng H.H., Miller A.A., Hart J.L. (2014). Hydrogen Sulfide Protects Endothelial Nitric Oxide Function under Conditions of Acute Oxidative Stress in Vitro. Naunyn Schmiedebergs Arch. Pharmacol..

[26] Whiteman M., Armstrong J.S., Chu S.H., Jia-Ling S., Wong B.-S., Cheung N.S., Halliwell B., Moore P.K. (2004). The Novel Neuromodulator Hydrogen Sulfide: An Endogenous Peroxynitrite “Scavenger”?. J. Neurochem..

[27] Koike S., Ogasawara Y., Shibuya N., Kimura H., Ishii K. (2013). Polysulfide Exerts a Protective Effect against Cytotoxicity Caused by T-Buthylhydroperoxide through Nrf2 Signaling in Neuroblastoma Cells. FEBS Lett..

[28] Mustafa A.K., Gadalla M.M., Sen N., Kim S., Mu W., Gazi S.K., Barrow R.K., Yang G., Wang R., Snyder S.H. (2009). H_2_S Signals through Protein S-Sulfhydration. Sci. Signal..

[29] Kimura H. (2021). Hydrogen Sulfide (H_2_S) and Polysulfide (H_2_Sn) Signaling: The First 25 Years. Biomolecules.

[30] Benchoam D., Cuevasanta E., Möller M.N., Alvarez B. (2019). Hydrogen Sulfide and Persulfides Oxidation by Biologically Relevant Oxidizing Species. Antioxidants.

[31] Cortese-Krott M.M., Kuhnle G.G.C., Dyson A., Fernandez B.O., Grman M., DuMond J.F., Barrow M.P., McLeod G., Nakagawa H., Ondrias K. (2015). Key Bioactive Reaction Products of the NO/H_2_S Interaction Are S/N-Hybrid Species, Polysulfides, and Nitroxyl. Proc. Natl. Acad. Sci. USA.

[32] Miyamoto R., Koike S., Takano Y., Shibuya N., Kimura Y., Hanaoka K., Urano Y., Ogasawara Y., Kimura H. (2017). Polysulfides (H_2_Sn) Produced from the Interaction of Hydrogen Sulfide (H_2_S) and Nitric Oxide (NO) Activate TRPA1 Channels. Sci. Rep..

[33] Baud L., Ardaillou R. (1986). Reactive Oxygen Species: Production and Role in the Kidney. Am. J. Physiol..

[34] Pfeilschifter J., Schwarzenbach H. (1990). Interleukin 1 and Tumor Necrosis Factor Stimulate CGMP Formation in Rat Renal Mesangial Cells. FEBS Lett..

[35] Hsu C.-N., Tain Y.-L. (2021). Gasotransmitters for the Therapeutic Prevention of Hypertension and Kidney Disease. Int. J. Mol. Sci..

[36] Zhang H., Zhao H., Guo N. (2021). Protective Effect of Hydrogen Sulfide on the Kidney (Review). Mol. Med. Rep..

[37] Fan Q.-L., Yang G., Liu X.-D., Ma J.-F., Feng J.-M., Jiang Y., Wang L.-N. (2013). Effect of Losartan on the Glomerular Protein Expression Profile of Type 2 Diabetic KKAy Mice. J. Nephrol..

[38] Sen U., Givvimani S., Abe O.A., Lederer E.D., Tyagi S.C. (2011). Cystathionine β-Synthase and Cystathionine γ-Lyase Double Gene Transfer Ameliorate Homocysteine-Mediated Mesangial Inflammation through Hydrogen Sulfide Generation. Am. J. Physiol. Cell. Physiol..

[39] Yuan P., Xue H., Zhou L., Qu L., Li C., Wang Z., Ni J., Yu C., Yao T., Huang Y. (2011). Rescue of Mesangial Cells from High Glucose-Induced over-Proliferation and Extracellular Matrix Secretion by Hydrogen Sulfide. Nephrol. Dial. Transplant..

[40] Xue H., Yuan P., Ni J., Li C., Shao D., Liu J., Shen Y., Wang Z., Zhou L., Zhang W. (2013). H_2_S Inhibits Hyperglycemia-Induced Intrarenal Renin-Angiotensin System Activation via Attenuation of Reactive Oxygen Species Generation. PLoS ONE.

[41] Kundu S., Pushpakumar S., Khundmiri S.J., Sen U. (2014). Hydrogen Sulfide Mitigates Hyperglycemic Remodeling via Liver Kinase B1-Adenosine Monophosphate-Activated Protein Kinase Signaling. Biochim. Biophys. Acta.

[42] Liu Y., Zhao H., Qiang Y., Qian G., Lu S., Chen J., Wang X., Guan Q., Liu Y., Fu Y. (2015). Effects of Hydrogen Sulfide on High Glucose-Induced Glomerular Podocyte Injury in Mice. Int. J. Clin. Exp. Pathol..

[43] Lee H.J., Feliers D., Mariappan M.M., Sataranatarajan K., Choudhury G.G., Gorin Y., Kasinath B.S. (2015). Tadalafil Integrates Nitric Oxide-Hydrogen Sulfide Signaling to Inhibit High Glucose-Induced Matrix Protein Synthesis in Podocytes. J. Biol. Chem..

[44] Szabo C. (2012). Roles of Hydrogen Sulfide in the Pathogenesis of Diabetes Mellitus and Its Complications. Antioxid. Redox Signal..

[45] Sun H.-J., Wu Z.-Y., Cao L., Zhu M.-Y., Liu T.-T., Guo L., Lin Y., Nie X.-W., Bian J.-S. (2019). Hydrogen Sulfide: Recent Progression and Perspectives for the Treatment of Diabetic Nephropathy. Molecules.

[46] Zhou X., Feng Y., Zhan Z., Chen J. (2014). Hydrogen Sulfide Alleviates Diabetic Nephropathy in a Streptozotocin-Induced Diabetic Rat Model. J. Biol. Chem..

[47] John A.M.S.P., Kundu S., Pushpakumar S., Fordham M., Weber G., Mukhopadhyay M., Sen U. (2017). GYY4137, a Hydrogen Sulfide Donor Modulates MiR194-Dependent Collagen Realignment in Diabetic Kidney. Sci. Rep..

[48] Qian X., Li X., Ma F., Luo S., Ge R., Zhu Y. (2016). Novel Hydrogen Sulfide-Releasing Compound, S-Propargyl-Cysteine, Prevents STZ-Induced Diabetic Nephropathy. Biochem. Biophys. Res. Commun..

[49] Paganelli F., Mottola G., Fromonot J., Marlinge M., Deharo P., Guieu R., Ruf J. (2021). Hyperhomocysteinemia and Cardiovascular Disease: Is the Adenosinergic System the Missing Link?. Int. J. Mol. Sci..

[50] Angelini A., Cappuccilli M.L., Magnoni G., Croci Chiocchini A.L., Aiello V., Napoletano A., Iacovella F., Troiano A., Mancini R., Capelli I. (2021). The Link between Homocysteine, Folic Acid and Vitamin B12 in Chronic Kidney Disease. G. Ital. Nefrol..

[51] Cordaro M., Siracusa R., Fusco R., Cuzzocrea S., Di Paola R., Impellizzeri D. (2021). Involvements of Hyperhomocysteinemia in Neurological Disorders. Metabolites.

[52] Kruger W.D. (2017). Cystathionine β-Synthase Deficiency: Of Mice and Men. Mol. Genet. Metab..

[53] Frosst P., Blom H.J., Milos R., Goyette P., Sheppard C.A., Matthews R.G., Boers G.J., den Heijer M., Kluijtmans L.A., van den Heuvel L.P. (1995). A Candidate Genetic Risk Factor for Vascular Disease: A Common Mutation in Methylenetetrahydrofolate Reductase. Nat. Genet..

[54] Yi F., Li P.-L. (2008). Mechanisms of Homocysteine-Induced Glomerular Injury and Sclerosis. Am. J. Nephrol..

[55] Sen U., Basu P., Abe O.A., Givvimani S., Tyagi N., Metreveli N., Shah K.S., Passmore J.C., Tyagi S.C. (2009). Hydrogen Sulfide Ameliorates Hyperhomocysteinemia-Associated Chronic Renal Failure. Am. J. Physiol. Renal Physiol..

[56] Sen U., Munjal C., Qipshidze N., Abe O., Gargoum R., Tyagi S.C. (2010). Hydrogen Sulfide Regulates Homocysteine-Mediated Glomerulosclerosis. Am. J. Nephrol..

[57] Majumder S., Ren L., Pushpakumar S., Sen U. (2019). Hydrogen Sulphide Mitigates Homocysteine-Induced Apoptosis and Matrix Remodelling in Mesangial Cells through Akt/FOXO1 Signalling Cascade. Cell. Signal..

[58] Pushpakumar S., Kundu S., Sen U. (2019). Hydrogen Sulfide Protects Hyperhomocysteinemia-Induced Renal Damage by Modulation of Caveolin and ENOS Interaction. Sci. Rep..

[59] Waz S., Heeba G.H., Hassanin S.O., Abdel-Latif R.G. (2021). Nephroprotective Effect of Exogenous Hydrogen Sulfide Donor against Cyclophosphamide-Induced Toxicity Is Mediated by Nrf2/HO-1/NF-ΚB Signaling Pathway. Life Sci..

[60] Pieretti J.C., Junho C.V.C., Carneiro-Ramos M.S., Seabra A.B. (2020). H_2_S- and NO-Releasing Gasotransmitter Platform: A Crosstalk Signaling Pathway in the Treatment of Acute Kidney Injury. Pharmacol. Res..

[61] Scammahorn J.J., Nguyen I.T.N., Bos E.M., Van Goor H., Joles J.A. (2021). Fighting Oxidative Stress with Sulfur: Hydrogen Sulfide in the Renal and Cardiovascular Systems. Antioxidants.

[62] Matthews J.R., Botting C.H., Panico M., Morris H.R., Hay R.T. (1996). Inhibition of NF-KappaB DNA Binding by Nitric Oxide. Nucleic. Acids. Res..

[63] Kelleher Z.T., Matsumoto A., Stamler J.S., Marshall H.E. (2007). NOS_2_ Regulation of NF-KappaB by S-Nitrosylation of P65. J. Biol. Chem..

[64] Sen N., Paul B.D., Gadalla M.M., Mustafa A.K., Sen T., Xu R., Kim S., Snyder S.H. (2012). Hydrogen Sulfide-Linked Sulfhydration of NF-ΚB Mediates Its Antiapoptotic Actions. Mol. Cell..

[65] Du J., Huang Y., Yan H., Zhang Q., Zhao M., Zhu M., Liu J., Chen S.X., Bu D., Tang C. (2014). Hydrogen Sulfide Suppresses Oxidized Low-Density Lipoprotein (Ox-LDL)-Stimulated Monocyte Chemoattractant Protein 1 Generation from Macrophages via the Nuclear Factor ΚB (NF-ΚB) Pathway. J. Biol. Chem..

[66] Yeh P.-Y., Li C.-Y., Hsieh C.-W., Yang Y.-C., Yang P.-M., Wung B.-S. (2014). CO-Releasing Molecules and Increased Heme Oxygenase-1 Induce Protein S-Glutathionylation to Modulate NF-ΚB Activity in Endothelial Cells. Free Radic. Biol. Med..

[67] Itoh K., Igarashi K., Hayashi N., Nishizawa M., Yamamoto M. (1995). Cloning and Characterization of a Novel Erythroid Cell-Derived CNC Family Transcription Factor Heterodimerizing with the Small Maf Family Proteins. Mol. Cell. Biol..

[68] Kobayashi M., Yamamoto M. (2005). Molecular Mechanisms Activating the Nrf2-Keap1 Pathway of Antioxidant Gene Regulation. Antioxid. Redox Signal..

[69] Um H.-C., Jang J.-H., Kim D.-H., Lee C., Surh Y.-J. (2011). Nitric Oxide Activates Nrf2 through S-Nitrosylation of Keap1 in PC12 Cells. Nitric. Oxide.

[70] Lee B.-S., Heo J., Kim Y.-M., Shim S.M., Pae H.-O., Kim Y.-M., Chung H.-T. (2006). Carbon Monoxide Mediates Heme Oxygenase 1 Induction via Nrf2 Activation in Hepatoma Cells. Biochem. Biophys. Res. Commun..

[71] D’Araio E., Shaw N., Millward A., Demaine A., Whiteman M., Hodgkinson A. (2014). Hydrogen Sulfide Induces Heme Oxygenase-1 in Human Kidney Cells. Acta. Diabetol..

[72] Wang B., Cao W., Biswal S., Doré S. (2011). Carbon Monoxide-Activated Nrf2 Pathway Leads to Protection against Permanent Focal Cerebral Ischemia. Stroke.

[73] Pilz R.B., Casteel D.E. (2003). Regulation of Gene Expression by Cyclic GMP. Circ. Res..

[74] Peters H., Wang Y., Loof T., Martini S., Kron S., Krämer S., Neumayer H.-H. (2004). Expression and Activity of Soluble Guanylate Cyclase in Injury and Repair of Anti-Thy1 Glomerulonephritis. Kidney Int..

[75] Wang Y., Krämer S., Loof T., Martini S., Kron S., Kawachi H., Shimizu F., Neumayer H.-H., Peters H. (2005). Stimulation of Soluble Guanylate Cyclase Slows Progression in Anti-Thy1-Induced Chronic Glomerulosclerosis. Kidney Int..

[76] Ren Y., D’Ambrosio M.A., Garvin J.L., Wang H., Carretero O.A. (2013). Mechanism of Inhibition of Tubuloglomerular Feedback by CO and CGMP. Hypertension.

[77] Morita T., Perrella M.A., Lee M.E., Kourembanas S. (1995). Smooth Muscle Cell-Derived Carbon Monoxide Is a Regulator of Vascular CGMP. Proc. Natl. Acad. Sci. USA.

[78] Bucci M., Papapetropoulos A., Vellecco V., Zhou Z., Pyriochou A., Roussos C., Roviezzo F., Brancaleone V., Cirino G. (2010). Hydrogen Sulfide Is an Endogenous Inhibitor of Phosphodiesterase Activity. Arterioscler. Thromb. Vasc. Biol..

[79] Zhou Z., Martin E., Sharina I., Esposito I., Szabo C., Bucci M., Cirino G., Papapetropoulos A. (2016). Regulation of Soluble Guanylyl Cyclase Redox State by Hydrogen Sulfide. Pharmacol. Res..

[80] Eberhardt W., Kunz D., Hummel R., Pfeilschifter J. (1996). Molecular Cloning of the Rat Inducible Nitric Oxide Synthase Gene Promoter. Biochem. Biophys. Res. Commun..

[81] Beck K.F., Sterzel R.B. (1996). Cloning and Sequencing of the Proximal Promoter of the Rat INOS Gene: Activation of NFkappaB Is Not Sufficient for Transcription of the INOS Gene in Rat Mesangial Cells. FEBS Lett..

[82] Sun W., Gao Y., Ding Y., Cao Y., Chen J., Lv G., Lu J., Yu B., Peng M., Xu H. (2019). Catalpol Ameliorates Advanced Glycation End Product-Induced Dysfunction of Glomerular Endothelial Cells via Regulating Nitric Oxide Synthesis by Inducible Nitric Oxide Synthase and Endothelial Nitric Oxide Synthase. IUBMB Life.

[83] Wang R. (2011). Signaling Pathways for the Vascular Effects of Hydrogen Sulfide. Curr. Opin. Nephrol. Hypertens..

[84] Schmidlin C.J., Rojo de la Vega M., Perer J., Zhang D.D., Wondrak G.T. (2020). Activation of NRF2 by Topical Apocarotenoid Treatment Mitigates Radiation-Induced Dermatitis. Redox Biol..

[85] Kanda H., Yamawaki K. (2020). Bardoxolone Methyl: Drug Development for Diabetic Kidney Disease. Clin. Exp. Nephrol..

[86] Yang W., Yang G., Jia X., Wu L., Wang R. (2005). Activation of KATP channels by H_2_S in rat insulin-secreting cells and the underlying mechanisms. J. Physiol..

